# Cardioprotective Effects of Curcumin-Nisin Based Poly Lactic Acid Nanoparticle on Myocardial Infarction in Guinea Pigs

**DOI:** 10.1038/s41598-018-35145-5

**Published:** 2018-11-09

**Authors:** Williams E. E. Nabofa, Oluwadamilola O. Alashe, Oyetunde T. Oyeyemi, Alfred F. Attah, Ademola A. Oyagbemi, Temidayo O. Omobowale, Adeolu A. Adedapo, Akinola R. A. Alada

**Affiliations:** 1 Department of Physiology, Bencarson (Snr) School of Medicine, Babcock University, Ilishan-Remo, Nigeria; 2 Department of Physiology, College of Medicine, University of Ibadan, Ibadan, Nigeria; 3 Department of Biological Sciences, University of Medical Sciences, Ondo, Ondo State Nigeria; 4 Department of Pharmacognosy, University of Ibadan, Ibadan, Nigeria; 5 Department of Veterinary Physiologv and Biochemistry, Faculty of Veterinary Medicine, University of Ibadan, Ibadan, Nigeria; 6 Department of Veterinary Medicine, Faculty of Veterinary Medicine, University of Ibadan, Ibadan, Nigeria; 7 Department of Veterinary Pharmacology and Toxicology, Faculty of Veterinary Medicine, University of Ibadan, Ibadan, Nigeria

## Abstract

Myocardial infarction (MI) is the most prevalent cause of cardiovascular death. A possible way of preventing MI maybe by dietary supplements. The present study was thus designed to ascertain the cardio-protective effect of a formulated curcumin and nisin based poly lactic acid nanoparticle (CurNisNp) on isoproterenol (ISO) induced MI in guinea pigs. Animals were pretreated for 7 days as follows; Groups A and B animals were given 0.5 mL/kg of normal saline, group C metoprolol (2 mg/kg), groups D and E CurNisNp 10 and 21 mg/kg respectively (n = 5). MI was induced on the 7^th^ day in groups B-E animals. On the 9^th^ day electrocardiogram (ECG) was recorded, blood samples and tissue biopsies were collected for analyses. Toxicity studies on CurNisNp were carried out. MI induction caused atrial fibrillation which was prevented by pretreatment of metoprolol or CurNisNp. MI induction was also associated with increased expressions of cardiac troponin I (CTnI) and kidney injury molecule-1 (KIM-1) which were significantly reduced in guinea pig’s pretreated with metoprolol or CurNisNp (P < 0.05). The LC_50_ of CurNisNp was 3258.2 μg/mL. This study demonstrated that the formulated curcumin-nisin based nanoparticle confers a significant level of cardio-protection in the guinea pig and is nontoxic.

## Introduction

Cardiovascular disease (CVD) remains a leading cause of disability and premature death globally. About 17.7 million people died as a result of CVD in 2015; contributing about 31% of all global death^1^. The world health organization (WHO) reported that of these deaths, about 7.4 million were due to coronary heart disease and 6.7 million were due to stroke^1^. Myocardial infarction (MI), more commonly known as heart attack is the most prevalent form of cardiovascular death in most countries of the world. MI occurs when one or more of the coronary arteries supplying blood to the heart are occluded, consequently depriving a section of the heart of oxygenated blood and nutrients, inevitably leading to necrosis of the myocardium^2^. MI is thus the progression to myocardial necrosis due to the critical imbalance between supply and demand of oxygen to the heart^3^. Catecholamine such as adrenaline and noradrenaline are capable of causing myocardial necrosis and increasing the progression of myocardial cell damage^4,5^. Many of the risk factors for MI are modifiable and thus in many cases may be preventable. A possible way of preventing MI maybe by dietary supplements.

Nutraceuticals is a term used for food derived isolates that have health benefits beside their actual function of providing nutrition. They are currently being employed in the prevention and treatment of diseases^6^. Examples of nutraceuticals include phytochemicals which are present in fruits and vegetables with potential health and physiological benefits particularly herbal polyphenol such as curcumin, beta-carotene, resveratrol and so on. Adverse side effects is a growing challenge in the use of therapeutic drugs. Over the years experimental studies have proven beyond doubt that nutraceuticals provide protection against diseases such as cardiovascular diseases, diabetes and cancer without adverse side effects^7^. The disease protecting ability of nutraceuticals is said to be due to their antioxidant, anti-inflammatory, antidiabetic and anticancer properties^7^. While nutraceuticals demonstrate these desirable properties they often lack stability, bioavailability and permeability. Thus the application of nanotechnology in the formulation of food supplements is of increasing interest especially in preventive medicine. Nanotechnology involves manipulating materials to a nanoscale with the purpose of creating new materials measuring between 1 and 100 nanometers. In the food and supplements industry there is a growing interest to create nanomaterial which allows nutrients to be more biologically effective by improving their transport, absorption and bioavailability in biological systems^8^.

Curcumin and nisin are components of natural sources^9,10^ with a wide range of biological activity^11–14^. Curcumin, a polyphenol responsible for the yellow color of the spice turmeric, has poor aqueous solubility and low bioavailability. However, at relatively low concentrations, i.p administration of curcumin has been reported to be an effective anti-inflammatory agent and exhibits cardioprotective effects^15^. The antioxidant properties of curcumin seem to be essential for its pleiotropic biological activities. Curcumin inhibits lipid peroxidation and effectively scavenges superoxide anion and hydroxyl radicals. It is also reported to interact with the mPTP^16^. The cardioprotective effect of curcumin against catecholamine-induced cardiotoxicity has been earlier reported^17^ and involves its antioxidant properties^18^. Moreover, curcumin inhibits nuclear factor-kB activation, protects cardiac cells against I/R injury^19^, and stabilizes the cytoskeleton through the increased expression of the heat shock protein Hsp27^20^.

Nisin is an antimicrobial peptide with probable anticancer properties evidenced in its ability to induce preferential apoptosis, cell cycle arrest, and reduce proliferation of HNSCC cells^12^. Although nothing is yet to be known about the cardioprotective effect of nisin, its broad spectrum of activities and use in food and dairy products preservation makes it a candidate compound that could be combined with curcumin in a nanoparticulate formulation for improved delivery in the treatment of cardiac-related defects. Despite the therapeutic uses of these compounds, they are commonly faced with problem of short half-life thus requiring frequent administration. Their extreme instability may cause easy degradation which results in poor pharmacokinetics, low bioavailability and pharmacological activity^21^. Therefore, development of a carrier that can maintain sustained release profile and avoid rapid degradation of the agents is essential for their effective therapeutic usage.

Biodegradable nanoparticles have been frequently used as drug delivery vehicles due to their grand bioavailability, better encapsulation, controlled release, and lesser toxicity^22^. Poly-D, L-lactic-co-glycolic acid (PLGA) and poly D, L-lactic acid (PLA) are one of the most successfully used biodegradable polymers for the development of nanoparticles. Their hydrolysis within the body produces non-toxic biodegradable metabolite monomers, lactic acid and glycolic acid^23^. Along with approval for use in humans by the US Food and Drug Administration^24^, these polymers are good candidates as carriers for drug delivery system^22,25^. The present study is thus designed to ascertain the cardioprotective effect of curcumin-nisin PLA encapsulated nanoparticles on isoproterenol induced myocardial infarction in guinea pigs.

## Results

### Properties of CurNisNp

**Figure 1:**
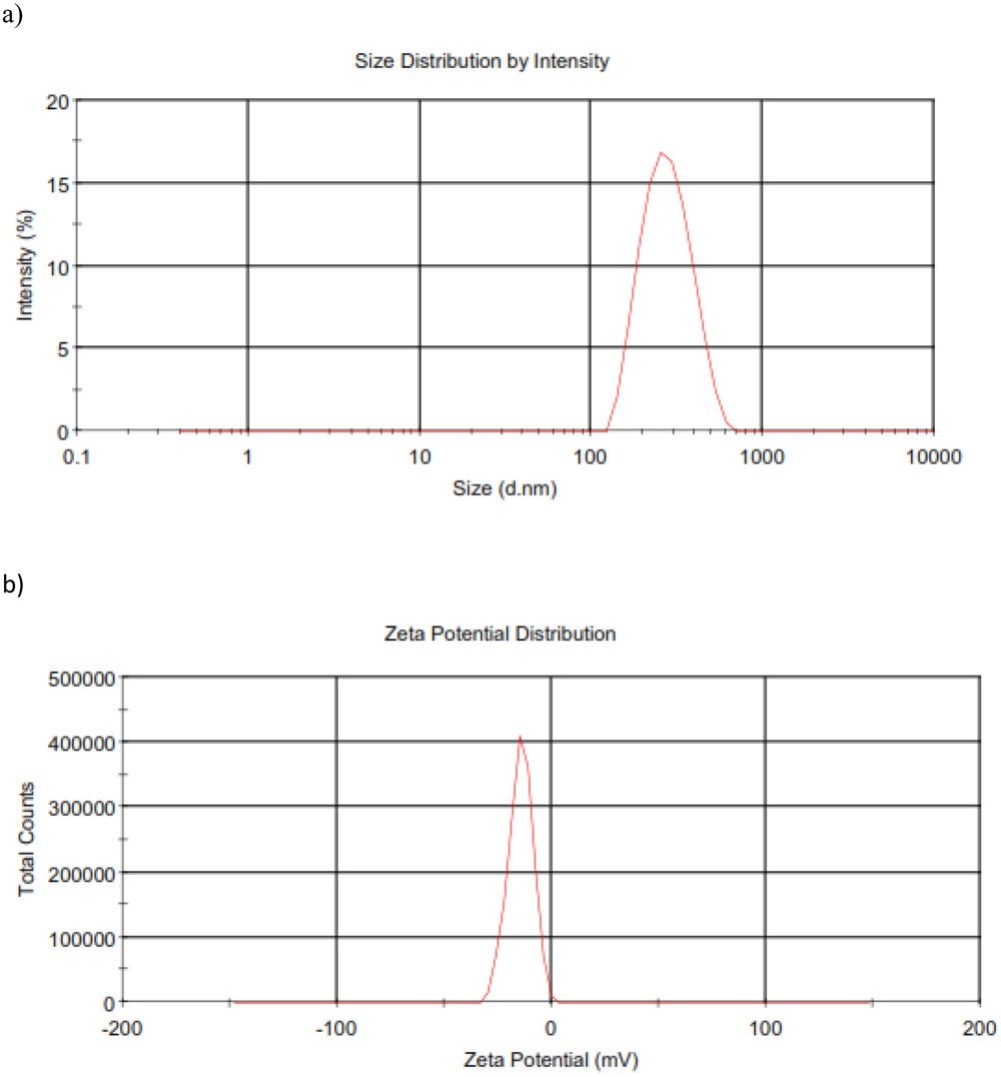
Properties of CurNisNp (**a**) Size distribution of CurNisNp (**b**) Zeta potential distribution of CurNisNp.

The nanoparticle size and zeta potential distributions are presented in Fig. 1 respectively. The mean size of CurNisNp used is 284.0 ± 17.9 nm.

### Effect of CurNisNp on hypertrophy index in hearts of MI induced guinea pigs

**Figure 2:**
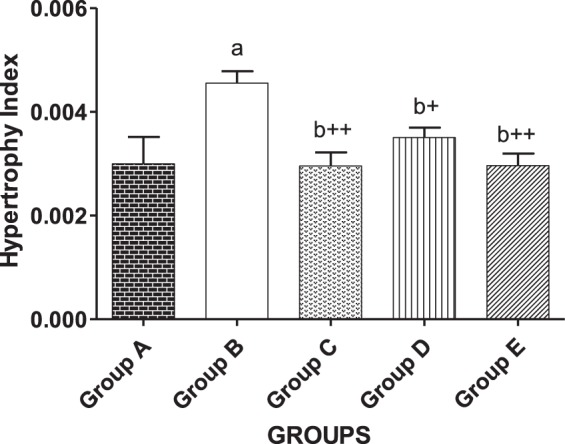
Effect of CurNisNp on hypertrophy index in MI induced guinea pigs. Note; data in bar chart are in Mean ± standard deviation. ^a^P < 0.05 compared with group A; ^b+^P < 0.01 compared with group B; ^b++^P < 0.001 compared with group B.

Administration of isoproterenol (10 mg/kg) to guinea pigs caused significant increases (P < 0.05) in hypertrophy index as seen in group B animals when compared to control (Fig. 2). Pretreatment of guinea pigs with metoprolol and CurNisNp (10 and 21 mg/kg) respectively prevented the significant increases in hypertrophy index due to administration of isoproterenol in guinea pigs.

### Histopathological examination

**Figure 3:**
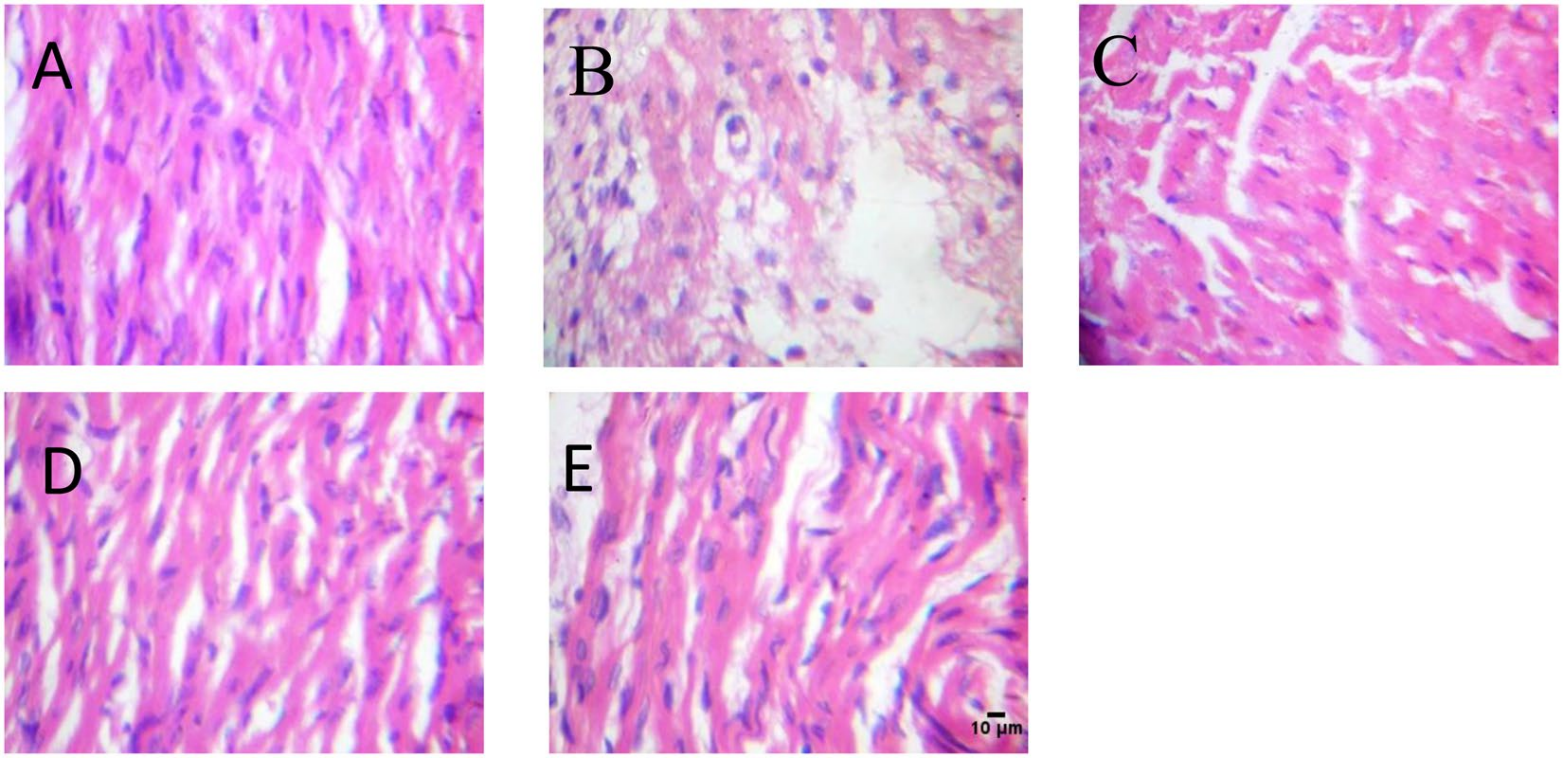
Histopathological changes of myocardial tissue (H&E); magnification ×400. (**A**) Control; (**B**) ISO alone; (**C**) ISO and metoprolol (**D**) ISO and CurNisNp (10 mg/kg) (**E**) ISO and CurNisNp (21 mg/kg). Group B showed myocardial cells necrosis, separation of cardiac myofibrillar and inflammatory cells infiltration due to ISO which were significantly reduced by pretreatment of metoprolol and CurNisNp respectively (Groups C–E).

Guinea pigs in the control group exhibited normal myocardial structure without any infarction (Fig. 3). However, isoproterenol-induced infarcted guinea pigs showed clear increase in myofibril thickness, necrosis, and loss of transverse striations compared to control group. Pretreatment of guinea pigs with metoprolol and CurNisNp (10 and 21 mg/kg) prevented the increase in myofibril thickness, necrosis, and loss of transverse striations due to isoproterenol treatment which were evident in the normal myocardial architectures of group C, D and E.

### ECG waves and intervals

**Figure 4:**
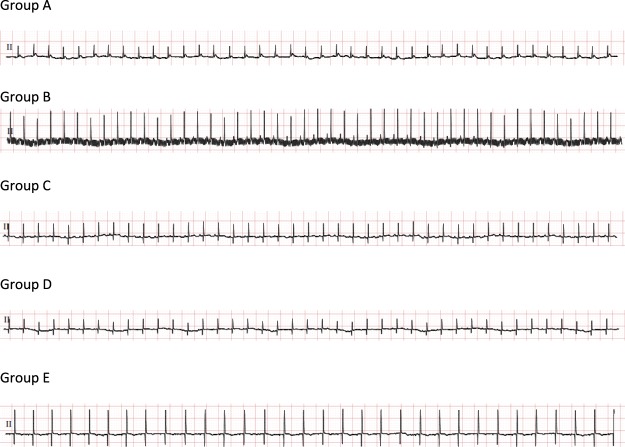
Effect of pretreatment of CurNisNp on ECG pattern in MI induced guinea pigs.

Electrocardiographic measurements for control and experimental guinea pigs are presented in Table 1 and Fig. 4. Control guinea pigs showed normal ECG pattern and normal heart rate, whereas guinea pigs treated with isoproterenol alone had significantly decreased (P < 0.05) heart rate, PR interval, increased QT/QTc interval and atrial fibrillation (Ramp; 1.27 ± 0.07 mV). Interestingly pretreatment with metoprolol or CurNisNp at both doses prevented the significant isoproterenol induced reduction in heart rate, PR-interval and increase in R-wave amplitude as seen in Table 1.

**Table 1:** Effect of pretreatment of CurNisNp on heart rate and ECG parameters in MI induced guinea pigs.

	HR (min)	P (ms)	PR int (ms)	QRS (ms)	QT int (ms)	QTc	R-amp (mV)
Group A	267.0 ± 7.8	25.0 ± 6.57	61.00 ± 2.56	32.33 ± 6.74	111.0 ± 21.03	234.0 ± 37.54	0.39 ± 0.12
Group B	237.0 ± 8.5^a++^	24.00 ± 7.00	52.50 ± 2.50^a++^	26.50 ± 1.50^a^	156.5 ± 9.50^a^	299.5 ± 5.50^a^	1.27 ± 0.07^a++^
Group C	255.0 ± 10^b+^	26.00 ± 2.00	62.50 ± 2.50^b++^	30.50 ± 3.50^b^	165.5 ± 6.50^a^	327.0 ± 1.00 ^a^	0.45 ± 0.11^b++^
Group D	249.5 ± 4.5^a,b^	22.50 ± 6.50	62.00 ± 1.00^b++^	27.00 ± 5.00	128.0 ± 12.0^b^	260.5 ± 21.50^b^	0.33 ± 0.07^b++^
Group E	253.5 ± 11.5^b^	24.00 ± 3.00	61.00 ± 1.00^b++^	35.00 ± 5.00^b+^	132.5 ± 21.5^b^	255.0 ± 28.00^b^	0.66 ± 0.31^b++^

Note; HR, heart rate; P, PR, QRS and QT are durations on the ECG wave form; QTc, corrected QT interval; R-amp, R-wave amplitude. The results in the table above are in Mean ± standard deviation of each group of five guinea pigs. ^a^P < 0.05 compared with group A; ^a++^P < 0.001 compared with group A; ^b^P < 0.05 compared with group B; ^b+^P < 0.01 compared with group B; ^b++^P < 0.001 compared with group B.

### Effect of pretreatment of CurNisNp on markers of oxidative stress and antioxidant defense system in MI induced guinea pigs

Animals treated with isoproterenol alone (group B) had significant increases (p < 0.05) in hydrogen peroxide tissue concentration in the heart and kidney which was associated with significant increases (p < 0.05) in cardiac and renal tissue MDA concentrations when compared with the control group as seen in Table 2. Pretreatment with CurNisNp at both doses (group D and E) prevented the significant increases in hydrogen peroxide and associated MDA increased concentration due to isoproterenol and this was similar in animals (group C) pretreated with metoprolol (Table 2). Also animals that were treated with isoproterenol alone (group B) had decrease total thiol and non-protein thiol which was significantly decreased at the renal tissue level when compared with control (p < 0.05). However when animals were pretreated with the higher dose of CurNisNp (group E) the significant reduction in renal total thiol and non-protein thiol due to isoproterenol treatment was significantly ablated as shown in Table 2.

**Table 2:** Effect of pretreatment of CurNisNp on markers of oxidative stress in MI induced guinea pigs.

	Group A	Group B	Group C	Group D	Group E
**Hydrogen peroxide (μmol/min mg protein)**					
Heart	38.35 ± 2.29	41.73 ± 4.41^a^	37.98 ± 1.61^b^	36.54 ± 2.14^b^	37.04 ± 2.27^b^
Kidney	42.06 ± 7.48	60.48 ± 5.76^a++^	44.39 ± 6.48^b++^	43.25 ± 7.10^b++^	38.50 ± 2.71^b++^
**Malondialdehyde (μmol/g tissue)**					
Heart	0.65 ± 0.03	0.78 ± 0.06^a++^	0.55 ± 0.03^a+,b++^	0.55 ± 0.03^a+,b++^	0.18 ± 0.01^a++,b++^
Kidney	0.50 ± 0.06	0.73 ± 0.19^a^	0.71 ± 0.19^a^	0.59 ± 0.10^b^	0.74 ± 0.04^a^
**Total thiol (nmole/mg protein)**					
Heart	56.68 ± 11.70	54.33 ± 6.87	60.14 ± 7.94	57.41 ± 9.34	53.01 ± 11.09
Kidney	41.46 ± 8.41	33.74 ± 3.69^a^	34.64 ± 5.32^a^	31.81 ± 3.41^a^	48.71 ± 7.99^b+^
**Non-protein thiol (nmole/mg protein)**					
Heart	8.43 ± 3.22	9.58 ± 1.64	12.82 ± 1.52^a,b+^	10.54 ± 2.48	15.93 ± 0.21^a,b^
Kidney	20.74 ± 5.87	15.72 ± 1.36^a^	31.20 ± 10.23^a,b++^	15.00 ± 4.53^a^	21.51 ± 6.058^b^

Note; results in the table above are in Mean ± standard deviation of each group of five guinea pigs. ^a^P < 0.05 compared with group A; ^a+^P < 0.01 compared with group A; ^a++^P < 0.001 compared with group A; ^b^P < 0.05 compared with group B; ^b+^P < 0.01 compared with group B; ^b++^P < 0.001 compared with group B.

SOD activity was significantly increased in cardiac tissues of animals treated with isoproterenol alone (goup B) but had significant decreased renal SOD activity. The reverse was the case with GSH activity in the same group of animals. Pretreatment with metopolol or CurNisNp before isoproterenol administration further increased the activity of SOD in cardiac tissues (p < 0.05) but significantly reversed the decrease in renal SOD activity due to isoproterenol treatment (Table 3). Pretreatment with metoprolol or CurNisNp significantly reversed the decrease in cardiac GSH activity and also reversed the significant increase in renal GSH activity due to isoproterenol administration (p < 0.05). GST activity was significantly decreased in both cardiac and renal tissues of animals treated with isoproterenol alone when compared with control animals (p < 0.05). Pretreatment with metoprolol or CurNisNp significantly reversed the decrease in both cardiac and renal tissue GST activity (Table 3).

**Table 3:** Effect of pretreatment of CurNisNp on antioxidant systems in MI induced guinea pigs.

	Group A	Group B	Group C	Group D	Group E
**SOD (units/mg protein)**					
Heart	7.59 ± 1.897	11.16 ± 1.17^a^	14.36 ± 1.14^a++,b+^	13.25 ± 1.71^a++,b^	16.32 ± 2.28^a++,b++^
Kidney	16.51 ± 1.67	13.75 ± 2.41^a^	15.97 ± 1.06^b^	17.61 ± 1.95^b^	16.81 ± 0.79^b^
**GST (μmole/mg protein)**					
Heart	0.031 ± 0.01	0.015 ± 0.01^a^	0.029 ± 0.003^b^	0.03 ± 0.01^b+^	0.029 ± 0.01^b^
Kidney	0.046 ± 0.01	0.023 ± 0.01^a^	0.041 ± 0.005^b^	0.042 ± 0.01^b^	0.04 ± 0.01^b^
**GSH (μmole/mg protein)**					
Heart	123.4 ± 3.82	119.1 ± 3.23^a+^	122.2 ± 2.57^b^	129.0 ± 6.06^a+,b++^	123.7 ± 5.17^b^
Kidney	194.1 ± 11.18	282.6 ± 33.53^a++,b++^	155.3 ± 15.47^a++,b++^	139.1 ± 9.72^a++,b++^	130.3 ± 5.35^a++,b++^

Note; results in the table above are in Mean ± standard deviation of each group of five guinea pigs. ^a^P < 0.05 compared with group A; ^a+^P < 0.01 compared with group A; ^a++^P < 0.001 compared with group A; ^b^P < 0.05 compared with group B; ^b+^P < 0.01 compared with group B; ^b++^P < 0.001 compared with group B.

### Expressions of CTnl and KIM-1

**Figure 5:**
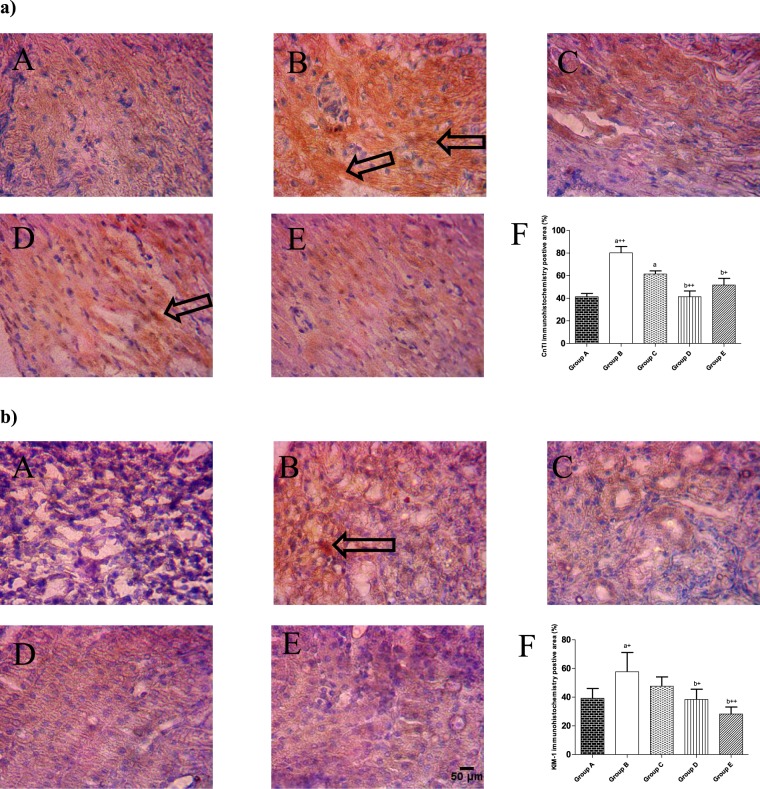
Immunohistochemical staining patterns of (**a**) cardiac troponin I (CnTI) in cardiac tissues, (**b**) Kidney Injury Molecule-1 in kidney tissues. (A) Control; (B) ISO alone; (C) ISO and metoprolol (D) ISO and CurNisNp (10 mg/kg) (E) ISO and CurNisNp (21 mg/kg). (F) Graphical representation of immunohistochemistry positive area. Intensity of staining is indicated with black arrows. Magnification ×100. ^a^P < 0.05 compared with group A; ^a+^P < 0.01 compared with group A; ^a++^P < 0.001 compared with group A; ^b+^P < 0.01 compared with group B; ^b++^P < 0.001 compared with group B.

Immunohistochemistry results showed that ISO administration increased the expression of CTnI and KIM-1 in guinea pig’s cardiac and kidney tissues respectively when compared with control (Fig. 5) and these increase in expressions of CTnI and KIM-1 due to ISO administration was significantly reduced in guinea pig’s pretreated with metoprolol or CurNisNp (10 or 21 mg/kg).

### Toxicity effect of CurNisNp

Brine Shrimp lethality activity of CurNisNp is shown in Table 4. CurNisNp showed 43% mortality at 1000 µg/mL concentration and its LC_50_ –value was 3258.2 mg/mL which is considered to be non-toxic. No mortality was found in negative control (distilled water) group.

**Table 4:** Percentage mortality of brine shrimp at different doses of CurNisNp.

Test Material	% Mortality under the concentration studied (μg/mL)					LC_50_ (μg/mL)	95%confidence Interval	Toxicity Profile
	1	10	100	500	1000			
CurNisNp	13	26	33	40	43	3258.2180	414.15–28705360.00	Non-toxic
K_2_Cr_2_O_7(positive control)_	20	47	83	100	100	8.9218	4.3953–16.1212	toxic
Sea water _(negative control)_	0	0	0	0	0	0	0	Non-toxic

## Discussion

The application of nanotechnology is of increasing interest in the formulation of nutraceuticals as this science provides grounds to improve supplements bioavailability, solubility, resistance to degradation by enzymes, improved blood circulation, reduced toxicity and limit their nonspecific uptake^26^. Though the present study is the first study to demonstrate the cardioprotective effect of CurNisNp in a mammalian model there are earlier reports of other types of formulated nanocurcumin particles in mammals with promising health effects^27^. Recent studies using CurNisNp had particularly reported its antiparasitic and molluscicidal activities^28,29^. The LC_50_ obtained in the present study shows that CurNisNp is a non-toxic nanoparticle and will be well tolerated in mammals. McLaughlin^30^ had earlier reported that results obtained with *Artemia salina* (brine shrimp) are quantitative and reproducible, and the activities parallel cytotoxicity. Thus our data demonstrates that CurNisNp still retains the non-toxic property of its parent material which is quite interesting because in nanotechnology an entirely new particle result from its parent material^22^. It is also worthy of note that this study is the first *in vitro* to demonstrate that CurNisNp is a non-toxic nanoparticle. Its non-toxicity is further supported by an *in vivo* toxicity mouse model; an observation which showed no significant toxicity on murine sperm cells^29^.

The use of guinea pigs in the present study affords a closer model to man than rats or mice since guinea pigs have ventricular action potential wave form close to those found in humans^31,32^, over 50% of their circulating lipids is LDL just like what obtains in man^33^ and they depend on external sources for ascorbic acid unlike rats^34,35^. Sustained sympathetic activation has been reported to be a hallmark of human heart failure, thus the use of isoproterenol, a beta adrenergic agonist, to induce cardiac damage in the present study further models what is common in human heart failure^36,37^.

Isoproterenol induced myocardial necrosis has been attributed to peroxidative damage since isoproterenol generates lipid peroxides^38^. Lipid peroxidation is a well-established mechanism for cardiac and renal cell damage and is a reliable indication of oxidative stress that leads to the pathogenesis of myocardial infarction^39^ and chronic kidney disease^40^.

The induction of myocardial infarction in guinea pigs by isoproterenol in the present study was associated with alterations in ECG patterns, cardiac remodeling, increase in MPO activity, oxidative stress, cardiac and renal tissue damage. Corneliu *et al*.^35^ had earlier reported that guinea pigs suffering from scurvy came down with myocardial infarction when administered with 10 mg/kg of isoproterenol. The present study further demonstrates that guinea pigs even without scurvy will come down with myocardial infarction when administered with 10 mg/kg of isoproterenol intra-peritoneally. Zhen *et al*.^41^ had also reported that single dose of 10 mg/kg of isoproterenol is capable of causing myocardial infarction in the Sprague-Dawley rat. Previous studies in other animal models have shown that isoproterenol impairs cardiomyocyte structure through oxidative stress and induction of cell apoptosis^42^ which also agrees with the results of the present study.

Pretreatment of guinea pigs with CurNisNp in the present study prevented isoproterenol induced myocardial infarction which was comparable to standard drug pretreatment. The mechanism by which CurNisNp prevents myocardial infarction includes its ability to increase the activity of the cardiac antioxidant defense which was accompanied with significantly lower levels of ROS, MDA, and CTnI expression in the heart when compared with isoproterenol alone treated animals. Cardiac troponins are tissue-specific biomarkers for cardiac damage^43^ and their significant increase in cardiac tissue expression have been reported to be a reliable predictor of cardiac death^44^. Thus the observed significant reduction in CTnl expression due to CurNisNp pretreatment further corroborates the fact that CurNisNp is cardioprotective. The ability of CurNisNp to protect renal tissues from oxidative damage is also demonstrated in the present study. As animal that were pretreated with CurNisNp before isoproterenol administration exhibited significant increases in their anti-oxidant defense and they did not show significant increase in renal MDA and hydrogen peroxide concentrations that was observed in animals treated with isoproterenol alone. Earlier studies suggest that significant increases in ROS in the kidney leads to renal inflammation, affecting renal structure and function, and subsequently leading to end stage renal disease (ESRD)^40,45–47^. KIM-1 is a type 1 transmembrane glycoprotein not detectable in healthy mammalian kidney tissues but inducible by ischemic and toxic insult. In acute and chronic renal failure the expression of KIM-1 has been reported to be significant^48,49^. Thus the very significant increase in KIM-1expression in the kidneys of animals treated with isoproterenol alone further corroborates the fact that isoproterenol administration is injurious to the kidneys of guinea pigs. It is noteworthy that pretreatment with CurNisNp significantly prevented KIM-1 induced expression by isoproterenol in the guinea pig renal tissue. The present study also demonstrates that a significant increase in renal oxidative stress is paralleled by a significant expression of KIM-1 and a significant reduction in renal oxidative stress is paralleled by a significant reduction in KIM-1 expression. In humans heart failure and chronic kidney disease often co-exist^50^, data from the present study also demonstrate that oxidative stress induced via activation of catecholamine pathway maybe capable of eliciting cardio-renal dysfunction.

Increase in MPO activity is associated with increased neutrophil infiltration^51^. Thus, the reduction in MPO activity by CurNisNp pretreatment in the present study portends that CurNisNp has anti-inflammatory properties. This is not surprising as one of the parent compounds curcumin has been widely reported for its anti-inflammatory properties^52,53^. The drug delivery system further potentiates these properties due to its controlled release and prolonged circulation in the biological system. Possible mechanisms by which CurNisNp reduces MPO activity may be due to its ability to decrease lipid peroxidation as observed in the present study.

In summary isoproterenol toxic effect in the guinea pig involves cardiac and renal tissue damage which is marked with increased expressions of CnTI and KIM-1 respectively. The present study also demonstrates that CurNisNp is a non-toxic nanoparticle substance that possesses protective effects on the guinea pig cardiac and renal tissues. The mechanism by which CurNisNp prevents cardiac and renal tissue damage involves its ability to enhance antioxidant defense and reduce ROS concentration.

## Materials and Method

### Ethical statement

All protocols in the present study was approved by Babcock University Research and Ethics Committee on Animal Care with the reference certificate number NHREC/17/12/2013 and are in adherence to international and national guidelines for the care and use of animals.

### Nanoparticle formulation and characterization

Curcumin-nisin poly-lactic acid nanoparticle (CurNisNp) which is a yellow crystalline powder comprising 35.0% composition by mass of the active compounds was prepared by the double emulsion-diffusion-evaporation method^28^. The nanoparticulate characterization by size, polydispersity index, zeta potential and *in vitro* release had earlier been reported^29^.

### Experimental design and animal treatment

Five month old adult male guinea pigs weighing 340–380 g were used. They were housed in plastic cages placed in a well-ventilated animal house and were given *ad libitum* access to guinea pig chow and subjected to natural photoperiod of 12 h light – 12 h dark cycle at an average room temperature of 23 °C and humidity of 60%. Guinea pigs were randomly divided into five groups of five animals. Animals were pretreated for 7 days as follows; Group A and B animals were administered 0.5 mL/kg of normal saline, group C animals metoprolol (2 mg/kg), group D and E CurNisNp 10 and 21 mg/kg respectively. MI was induced on the 7^th^ day in groups II-V animals. On the 9^th^ day ECG was recorded and blood samples were collected by cardiac puncture before animals were sacrificed. Heart and kidney tissue biopsies were collected on dry ice for biochemical, histological and immunohistochemical analysis. Toxicity studies of CurNisNp were also carried out with the aid of brine shrimp lethality test.

### CurNisNp experimental dose

60 mg/kg of curcumin had earlier been reported to be the effective dose for health promoting antioxidant and anti-inflammatory activities in rodents^54,55^. Since CurNisNp is 35.0% composition by mass of curcumin, the nanoformulation equivalent of 60 mg/kg of curcumin will be 21 mg/Kg of CurNisNp. Thus in the present study the cardioprotective effect of 21 mg/kg of CurNisNp and a lower dose of 10 mg/kg was determined.

### Induction of myocardial infarction

ISO was dissolved in saline and a single dose of 10 mg/Kg was administered to guinea pigs via subcutaneous injection to induce myocardial infarction^56,57^. Solutions were prepared fresh and used within 30 minutes of preparation. Animals were sacrificed 48 hours after the single dose of ISO was induced in guinea pigs as previously described by Zeana *et al*. and Zhen *et al*. B.

### Electrocardiography

Standard lead II electrocardiogram was recorded in guinea pigs immobilized with xylazine–ketamine combination using a 6/7-lead ECG machine (EDAN VE-1010, Shanghai, China). The machine was calibrated at 20 mm/mV paper speed and 50 mm/s paper speed. From the electrocardiogram, parameters such as heart rate, PR interval, QRS wave duration, R-wave amplitude, and QT/QTc values were determined.

### Serum preparation and isolation of post-mitochondrial fraction

About 3 mL of blood was collected from the heart of animals by cardiac puncture into plain sample bottles during xylazine–ketamine induced anesthesia after which animals were sacrificed by cervical dislocation. The blood was centrifuged at 4000 rpm for 15 min to obtain the serum. Kidneys and hearts of animals were harvested on ice, rinsed, and homogenized in aqueous potassium buffer (0.1 M, pH 7.4) and the homogenate centrifuged at 10,000 rpm (4 °C) for 10 min to obtain the supernatant fraction.

### Biochemical assays

Determination of protein concentration was done by Biuret method as described by Gornal *et al*.^56^. Hydrogen peroxide (H_2_O_2_) generation was determined with the aid of spectrophotometry at 560 nm as described by Wolff^57^. Lipid peroxidation was assessed by estimating malondialdehyde (MDA) using the method of Varshney and Kale^30^. MDA tissue concentration was quantified with a molar extinction coefficient of 1.56 × 10^5^ M^−1^cm^−1^ and expressed as micromoles per gram of tissue. Superoxide dismutase (SOD) activity was determined by measuring the inhibition of the auto-oxidation of epinephrine at pH 7.2 at 30 °C as described by Misra and Fridovich^58^ and modified by Oyagbemi *et al*.^59^. Briefly, 100 mg of epinephrine was dissolved in 100 mL distilled water and acidified with 0.5 mL concentrated hydrochloric acid. Then, 0.01 mL of each sample was added to 2.5 mL of 0.05 mol/L carbonate buffer (pH 10.2), followed by the addition of 0.3 mL of 0.3 mmol/L epinephrine. The increase in absorbance at 480 nm was monitored every 30 s for 150 s. One unit of SOD activity represents the amount of SOD necessary to cause 50% inhibition of the oxidation of adrenaline to adrenochrome during 1 min. The concentration of GSH was determined at 412 nm using the method described by Jollow *et al*.^60^. Glutathione-S-transferase (GST) activity was estimated by the method of Habig *et al*.^61^ using 1-chloro-2, 4-dinitrobenzene (CDNB) as substrate. Total thiol and non-protein thiol (NPT) concentrations were determined as described by Ellman^62^. Serum myeloperokidase (MPO) activity was determined according to the method of Xia and Zweier^63^.

### Measurement of hypertrophy index

Animals were weighed before their death and recorded in grams. Isolated guinea pig heart was also weighed upon death, and the wet weight of the heart was recorded in milligrams. Hypertrophy index was calculated as a ratio of the animal heart weight to animal body weight.

### Immunohistochemistry of cardiac troponin I (CTnI) and kidney injury molecule-1 (KIM-1)

Immunohistochemistry of paraffin-embedded heart and kidney tissues was performed after the tissues were fixed with 10% buffer formalin based on the methods described by Oyagbemi *et al*.^64^. Briefly, paraffin sections were melted at 60 °C in the oven. Dewaxing of the samples in xylene was followed by passage through graded ethanol (100–80%). Peroxidase quenching with 1% H_2_O_2_/methanol was followed by antigen retrieval performed by microwave heating in 0.01 mol/L citrate buffer (pH 6.0) to boil. All the sections were blocked in normal goat serum (10%, HistoMark, KPL, Gaithersburg, Maryland, USA) and probed with anti-CTnl and anti-KIM-1 antibodies, as appropriate (Bioss, San Diego, California, USA), 1:200 overnight at room temperature. Detection of bound antibody was carried out using biotinylated (goat anti-rabbit, 2.0 μg/mL) secondary antibody and subsequently, streptavidin peroxidase (horseradish peroxidase-streptavidin) according to manufacturer’s protocol (HistoMark, KPL, Gaithersburg, Maryland, USA). The reaction product was enhanced with diaminobenzidine (DAB, Amresco, USA) for 2–3 min and counterstained with high definition hematoxylin (Enzo, New York, USA), with subsequent dehydration in ethanol. The slides were covered with coverslips and sealed with resinous solution. The immune-reactive positive expression of CTnl and KIM-1 intensive regions were viewed starting from low magnification on each slide then with 400× magnifications using a photo microscope (Olympus) and a digital camera (Toupcam; Touptek Photonics, Zhejiang, China). The measurement of immune-reactive positive expression of CTn1 and KIM-1 were carried out digitally using quantification software (ImageJ 1.48 v; National Institutes of Health, Bethesda, MD, USA). Five (5) photomicrographs were analyzed per group for each parameter.

### Histopathology

Biopsies of cardiac and renal tissues were collected in 10% buffered formalin (pH 7.4, 25 °C) and kept for a minimum of 2 days for proper fixation. These tissues were processed and embedded in paraffin wax. Sections of 5–6 μm in thickness were made and stained with hematoxylin-eosin^65^ and were examined by a clinical pathologist.

### Toxicity test for CurNisNp

CurNisNp was screened for toxicity using larvae (nauplii) of *Artemia Salina* (brine shrimp) as described by Solis *et al*.^66^. The CurNisNp was reconstituted in saltwater for brine shrimp lethality assay. The test was performed in triplicate using well calibrated 15 mL eppendorff tubes, with nanoparticle concentrations of 1000, 500, 100, 10 and 1 mg/mL. *Artemia* eggs were incubated for 36 h with natural seawater. The nauplii were collected and brought into contact with the test substances. After 24 h of incubation at room temperature in the light, the number of surviving nauplii in each well was determined with the aid of a hand lens. Sea water without the test substance was used as negative control while potassium dichromate served as positive control. The 50% lethal concentrations (LC_50_) of CurNisNp was determined by Finney’s probit analysis. The mean ± standard deviation of the mean LC_50_ was calculated from three independent experiments. CurNisNp dilutions that did not show toxicity were considered nontoxic^66,67^.

### Statistical analyses

Statistical analyses were carried out using GraphPad Prism software (version 5.00). One-way analysis of variance (ANOVA) was used to compare significant differences in parameters measured amongst experimental groups. Tukey’s post-hoc test was further used to compare significant differences within groups. Student’s t test was used to test significance between two experimental groups. P values < 0.05 were considered statistically significant.

### Ethical approval

All protocols in the present study was approved by Babcock University Research and Ethics Committee on Animal Care with the reference certificate number NHREC/17/12/2013 and are in adherence to international and national guidelines for the care and use of animals.

## References

[1] World health organization (WHO) fact sheet http://www.who.int/mediacentre/factsheets/fs317/en/ (2017).

[2] De Bono, D. P. & Boon, N. A. *Diseases of the cardiovascular system*. *In: Davidson’s Principles and Practice of Medicine*. (eds Edwards, C.R.W. & Boucheir, I.A.S) 249–340 (Hong Kong: Churchill Livingstone, 1992).

[3] Zhu YZ (2000). Effect of losartan on hemodynamic parameters- and angiotensin receptor mRNA levels of rat heart after myocardial infarction. J. Renin Angioten. Aldoster. Syst..

[4] Waldenstrom AP, Hjalmarson AC, Thornell L (1978). A possible role of noradrenaline in the development of myocardial infarction. Am. Heart J..

[5] Bacaner M, Brietenbucher J, LaBree J (2004). Prevention of ventricular fibrillation, acute myocardial infarction (myocardial necrosis), heart failure, and mortality by bretylium: is ischemic heart disease primarily adrenergic casdiovascular disease?. Am. J. Ther..

[6] Brower V (1998). Nutraceuticals: Poised for a healthy slice of the healthcare market?. Nat. Biotechnol..

[7] Gupta S, Chauhan D, Mehla K, Sood P, Nair A (2010). An overview of neutraceuticals: Current scenario. J. Basic Clin. Pharm..

[8] Acosta E (2009). Bioavailability of nanoparticles in nutrient and nutraceutical delivery. Cur. Opinion in Colloid and Interface Sci..

[9] Chainani-Wu N (2003). Safety and anti-inflammatory activity of curcumin: a component of tumeric (*Curcuma longa*). J. Altern. Complement Med..

[10] Jones, E., Salin, V. & Williams, G. W. Nisin and the market for commercial bacteriocins. (Consumer and Product Research Report) CP-01-05. (TAMRC, 2005).

[11] Ishita C, Kaushik B, Uday B, Ranajit KB (2004). Turmeric and curcumin: Biological actions and medicinal applications. Curr. Sci..

[12] Joo NE, Ritchie K, Kamarajan P, Miao D, Kapila YL (2012). Nisin, an apoptogenic bacteriocin and food preservative, attenuates HNSCC tumorigenesis via CHAC1. Cancer Med..

[13] Allam G (2009). Immunomodulatory effects of curcumin treatment on murine schistosomiasis mansoni. Immunobiol..

[14] Magalhães LG (2009). *In vitro* schistosomicidal activity of curcumin against *Schistosoma mansoni* adult worms. Parasitol Res..

[15] Wongcharoen W, Phrommintikul A (2009). The protective role of curcumin in cardiovascular diseases. Int. J. of Cardiology..

[16] Morin D, Barthelemy S, Zini R, Labidalle S, Tillement JP (2001). Curcumin induces the mitochondrial permeability transition pore mediated by membrane protein thiol oxidation. FEBS Lett..

[17] Nirmala C, Puvanakrishnan R (1996). Protective role of curcumin against isoproterenol induced myocardial infarction in rats. Mol. Cell Biochem..

[18] Manikandan P (2004). Curcumin modulates free radical quenching in myocardial ischaemia in rats. Int. J. Biochem. Cell Biol..

[19] Fiorillo C (2008). Curcumin protects cardiac cells against ischemia-reperfusion injury: effects on oxidative stress, NF-kappaB, and JNK pathways. Free Radic. Biol. Med..

[20] Tanwar V, Sachdeva J, Golechha M, Kumari S, Arya DS (2010). Curcumin protects rat myocardium against isoproterenol-induced ischemic injury: attenuation of ventricular dysfunction through increased expression of Hsp27 along with strengthening antioxidant defense system. J. Cardiovasc. Pharmacol..

[21] Burgos-Moron E, Calderon-Montano JM, Salvador J, Robles A, Lopez-Lazaro M (2010). The dark side of curcumin. Int. J. Cancer..

[22] Nguyen HT (2015). Enhancing the *in vitro* anti-cancer efficacy of artesunate by loading into poly D,L-lactide-co-glycolide (PLGA) nanoparticles. Arch. Pharm. Res..

[23] Kumari A, Yadav SK, Yadav SC (2010). Biodegradable polymeric nanoparticles based drug delivery systems. Coll. Surf. B: Biointer..

[24] Jain RA (2000). The manufacturing techniques of various drug loaded biodegradable poly (lactide-co-glycolide) (PLGA) devices. Biomaterials..

[25] Pradhan R (2013). Docetaxel loaded polylactic acid-co-glycolic acid nanoparticles: formulation, physicochemical characterization and cytotoxicity studies. J. Nanosci. Nanotechnol..

[26] Freitas RA (2005). What is nanomedicine?. Nanomedicine: Nanotechnology, Biol. and Med..

[27] Ghalandarlaki N, Alizadeh AM, Ashkani-Esfahani S (2014). Nanotechnology-Applied Curcumin for different diseases therapy. Hindawi Publishing Corp. BioMed. Res. Int..

[28] Omobhude ME, Morenikeji OA, Oyeyemi OT (2017). Molluscicidal activities of curcumin-nisin polylactic acid nanoparticle on Biomphalaria pfeifferi. Plos Neglected Tropical Dis..

[29] Oyeyemi Oyetunde, Adegbeyeni Odunayo, Oyeyemi Ifeoluwa, Meena Jairam, Panda Amulya (2018). In vitro ovicidal activity of poly lactic acid curcumin-nisin co-entrapped nanoparticle against Fasciola spp. eggs and its reproductive toxicity. Journal of Basic and Clinical Physiology and Pharmacology.

[30] Varshney R, Kale RK (1990). Effects of calmodulin antagonists on radiation-induced lipid peroxidation in microsomes. Int. J. of Radiation Biol..

[31] Watanabe T, Rautaharju PM, McDonald TF (1985). Ventricular action potentials, ventricular extracellular potentials, and the ECG of guinea-pig. Circ. Res..

[32] Soltysinska E, Olesen SP, Osadchii OE (2011). Myocardial structural, contractile and electrophysiological changes in the guinea-pig heart failure model induced by chronic sympathetic activation. Exp. Physiol..

[33] Fernandez ML, Volek JS (2006). Guinea pigs: a suitable animal model to study lipoprotein metabolism, atherosclerosis and inflammation. Nutr. Metab..

[34] Burns JJ, Burch HB, King CG (1951). The metabolism of l-C”-ascorbic acid in guinea pigs. J. Biol. Chem..

[35] Corneliu Z., Desideriu L. & Constantinescu S. Isoproterenol Induced Myocardial Infarction in Guinea Pigs with Scurvy. Argentine Federation of Cardiology (2001).

[36] Cohn JN (1984). Plasma norepinephrine as a guide to prognosis in patients with chronic congestive heart failure. N. Engl. J. Med..

[37] Kaye DM (1995). Adverse consequences of high sympathetic nervous activity in the failing human heart. J. Am. Coll. Cardiol..

[38] Blasig IE, Blasig R, Lowe H (1984). Myocardial lipid peroxidation during isoproterenol-induced blood flow reduction in rat myocardium. Biomedica Biochimica Acta..

[39] Mohanty I (2004). Mechanisms of cardioprotective effect of Withania somnifera in experimentally induced myocardial infarction. Basic and Clin. Pharm. & Toxicol..

[40] Cachofeiro Victoria, Goicochea Marian, de Vinuesa Soledad García, Oubiña Pilar, Lahera Vicente, Luño José (2008). Oxidative stress and inflammation, a link between chronic kidney disease and cardiovascular disease. Kidney International.

[41] Zhen EY (2007). Quantification of heart fatty acid–binding protein as a biomarker for drug-induced cardiac and musculoskeletal necroses. Proteomics.

[42] Daoud Amal, Ben mefteh Fedia, Mnafgui Kais, Turki Mouna, Jmal Salwa, Ben amar Rawdha, Ayadi Fatma, ElFeki Abdelfattah, Abid Leila, Rateb Mostafa E., belbahri Lassaad, Kadri Adel, Gharsallah Neji (2017). Cardiopreventive effect of ethanolic extract of Date Palm Pollen against isoproterenol induced myocardial infarction in rats through the inhibition of the angiotensin-converting enzyme. Experimental and Toxicologic Pathology.

[43] Adamcová M (2005). Troponin as a marker of myocardiac damage in drug-induced cardiotoxicity. Expert Opin. Drug Safety.

[44] Acikel M (2005). Protective effects of dantrolene against myocardial injury induced by isoproterenol in rats: biochemical and histological findings. Int. J. Cardiol..

[45] Forbes JM, Coughlan MT, Cooper ME (2008). Oxidative stress as a major culprit in kidney disease in diabetes. Diabetes.

[46] Shalamanova L, McArdle F, Amara AB, Jackson MJ, Rustom R (2007). Albumin overload induces adaptive responses in human proximal tubular cells through oxidative stress but not via angiotensin II type 1receptor. Am. J. Physiol. Renal Physiol..

[47] Kim J, Seok YM, Jung KJ, Park KM (2009). Reactive oxygen species/oxidative stress contributes to progression of kidney fibrosis following transient ischemic injury in mice. Am. J. Physiol. Renal Physiol..

[48] Ichimura T (1998). Kidney injury molecule-1 (KIM-1), a putative epithelial cell adhesion molecule containing a novel immunoglobulin domain, is up-regulated in renal cells after injury. J. Biol. Chem..

[49] Vaidya VS, Ramirez V, Ichimura T, Bodadilla NA, Bonventre JV (2006). Urinary Kidney injury molecule −1: a sensitive quantivative biomarker for early detection of kidney tubular injury. Am. J. Physiol..

[50] Adams KF (2005). ADHERE Scientific Advisory Committee and Investigators Characteristics and outcomes of patients hospitalized for heart failure in the United States: rationale, design, and preliminary observations from the first 100 000 cases in the Acute Decompensated Heart Failure National Registry (ADHERE). Am. Heart J..

[51] Schindhelm RK, Zwan LP, Teerlink T, Scheffer PG (2009). Myeloperoxidase: A Useful Biomarker for Cardiovascular Disease Risk Stratification?. Clin. Chem..

[52] Ferreira VH, Nazli A, Dizzell SE, Mueller K, Kaushic C (2015). The anti-inflammatory activity of curcumin protects the genital mucosal epithelial barrier from distruption and blocks replication of HIV-1 and HSV-2. PLoS One. 9.

[53] Edwards RL (2017). The anti-inflammatory of curcumin is mediated by its oxidative metabolites. J. of Biological Chem..

[54] Izem-Meziane M (2012). Catechlamine-induced cardiac mitochondrial dysfunction and mPTP opening protective effect of curcumin. Am. J. Physiol. Heart Circ. Physiol..

[55] Roshankhah SH (2017). Effects of curcumin on sperm parameters abnormalities induced by morphine in rat. J. of Medical and Biomed. Sciences.

[56] Gornall AG, Bardawill CJ, David MM (1949). Determination of serum proteins by means of Biuret reaction. J. Biol. Chem..

[57] Wolff SP (1994). Ferrous ion oxidation in the presence of ferric ion indicator xylenol orange for measurement of hydroperoxides. Methods Enzymol..

[58] Misra H, Fridovich I (1972). The Role of Superoxide Anion in the Autooxidation of Epinephrine and a Simple Assay for Superoxide Dismutase. J. Biol. Chem..

[59] Oyagbemi AA (2015). Lack of reversal of oxidative damage in renal tissues of lead acetate-treated rats. Environ. Toxicol..

[60] Jollow DJ, Mitchell JR, Zampaglione N, Gillette JR (1974). Bromobenzeneinduced liver necrosis. Protective role of glutathione and evidence for 3, 4–bromobenzene oxide as the hepatotoxic metabolite. Pharm..

[61] Habig WH, Pabst MJ, Jakoby WB (1974). Glutathione S-transferases. The first enzymic step in mercapturic acid formation. J. Biol. Chem..

[62] Ellman GL (1959). Tissue sulfhydryl groups. Arch. Biochem. Biophys..

[63] Xia Y, Zweier JL (1997). Measurement of myeloperoxidase in leukocyte-containing tissues. Anal. Biochem..

[64] Oyagbemi AA (2017). Sodium fluoride induces hypertension and cardiac complications through generation of reactive oxygen species and activation of nuclear factor kappa beta. Environ. Toxicol..

[65] Drury, R.A., Wallington, E.A., & Cancerson, R. Carlton’s histopathological techniques. 4th edition (Oxford University Press, 1976).

[66] Solís PN, Wright CW, Anderson MA, Gupta MP, Phillipson JD (1993). A microwell cytotoxicity assay using Artemia salina (brine shrimp). Planta Med..

[67] Anderson JE, Goetz CM, McLaughlin JL (1991). A blind comparison of simple bench top bioassays and human tumor cell cytotoxicities as antitumor prescreens. Phytochem. Anal..

